# Targeted Mass Spectrometry Analysis of *Clostridium perfringens* Toxins

**DOI:** 10.3390/toxins11030177

**Published:** 2019-03-23

**Authors:** Miloslava Duracova, Jana Klimentova, Alena Myslivcova Fucikova, Lenka Zidkova, Valeria Sheshko, Helena Rehulkova, Jiri Dresler, Zuzana Krocova

**Affiliations:** 1 Faculty of Military Health Sciences, University of Defense in Brno, Třebešská 1575, CZ-500 01 Hradec Králové, Czech Republic; miloslava.duracova@unob.cz (M.D.); alena.myslivcovafucikova@unob.cz (A.M.F.); murfara@gmail.com (L.Z.); valeria.sesko@unob.cz (V.S.); helena.rehulkova@unob.cz (H.R.); zuzana.krocova@unob.cz (Z.K.); 2 Department of Biology, Faculty of Science, University of Hradec Kralove, Hradecká 1285, CZ-500 03 Hradec Kralove, Czech Republic; 3 Military Health Institute, Military Medical Agency, Tychonova 1, CZ-160 00 Prague 6, Czech Republic; jiri.dresler@gmail.com

**Keywords:** mass spectrometry, PRM, *Clostridium perfringens*, protein toxin, epsilon toxin

## Abstract

Targeted proteomics recently proved to be a technique for the detection and absolute quantification of proteins not easily accessible to classical bottom-up approaches. Due to this, it has been considered as a high fidelity tool to detect potential warfare agents in wide spread kinds of biological and environmental matrices. *Clostridium perfringens* toxins are considered to be potential biological weapons, especially the epsilon toxin which belongs to a group of the most powerful bacterial toxins. Here, the development of a target mass spectrometry method for the detection of *C. perfringens* protein toxins (alpha, beta, beta2, epsilon, iota) is described. A high-resolution mass spectrometer with a quadrupole-Orbitrap system operating in target acquisition mode (parallel reaction monitoring) was utilized. Because of the lack of commercial protein toxin standards recombinant toxins were prepared within *Escherichia coli*. The analysis was performed using proteotypic peptides as the target compounds together with their isotopically labeled synthetic analogues as internal standards. Calibration curves were calculated for each peptide in concentrations ranging from 0.635 to 1101 fmol/μL. Limits of detection and quantification were determined for each peptide in blank matrices.

## 1. Introduction

*Clostridium perfringens* is a gram-positive, anaerobic, spore-forming bacterium spread throughout the environment, especially in soil. It is a common agent in the gastrointestinal tracts of healthy humans and animals. At the same time, this agent is one of the most significant producers of toxins among all known bacteria. This expressive toxicity is due to the bacteria’s ability collectively to produce different protein toxins and/or enzymes with diverse modes of action. 

The bacterium was first identified at the end of the 19th century, when autopsies were performed on cancer and tuberculosis patients, 8 h postmortem. Gas bubbles were observed perfused throughout the body and appeared particularly within blood vessels. Gas (carbon dioxide plus hydrogen) and organic acids (e.g., acetic, butyric, and lactic) are common byproducts of anaerobic metabolism of *C. perfringens* [1]. Other toxins from the pathogenic *Clostridium* genus include such dangerous agents as tetanus and botulinum neurotoxins.

Historically, the species *C. perfringens* was classified into five toxinotypes (A–E) according to the presence and combination of the main toxins that the bacterium produces: alpha, beta, epsilon, or iota. The newly updated toxinotyping scheme incorporates two new toxinotypes (F and G) with the new combination of the main toxins: alpha, beta, epsilon, enterotoxin, NetB [2]. 

However, to date it is known that *C. perfringens* produces as many as 20 toxins: alpha (CPA), beta (CPB), epsilon (ETX), iota (CPI), enterotoxin (CPE), theta/perfringolysin O (PFO), beta-2 (CPB2), TpeL, NetB, BecA/B, NetE, NetF, NetG, NanI, NanJ, kappa, mu, lambda, clostripain, and delta [3,4].

Under natural conditions, this bacterium is responsible for local outbreaks of food poisoning associated with eating contaminated food that was improperly heat treated. The bacterium is also a major cause of gas gangrene, a disease associated with wound infection that has a fatal prognosis in cases of delayed treatment. In the absence of early radical surgery, antibiotic therapy and (if available) hyperbaric treatment, the infection leads to the spread of toxins in the body causing shock, coma, and death. Due to the strength of the toxins produced, *C. perfringens* constitutes a possible source for the production of biological weapons. It could potentially be used to induce outbreaks of food poisoning and, through disseminated contamination by spores, increased morbidity of gas gangrene in injured individuals. *C. perfringens* types B and D produce epsilon toxin (ETX), which is considered to be the third most powerful bacterial toxin [5]. Because the toxin can be dispersed as an aerosol and methods are lacking for the treatment and preventing its poisoning actions, it is a potential tool for bioterrorism. The United States Department of Agriculture and Centers for Disease Control and Prevention have previously classified ETX as a select agent, similarly like other protein toxins such as ricin and staphylococcal enterotoxin B. In a modification of the select agents list from December 2012, ETX was removed from this list [6]. In France, however, ETX is still classified as a potential biological weapon requiring special authorization for laboratory work from the Agence nationale de sécurité du médicament et des produits de santé (ANSM) [7]. In any case, the other toxins produced by *C. perfringens* are not mentioned in any other list represent significant risk for human beings or in the veterinary area.

Traditionally, in vivo and in vitro bioassay and immunoassay-based methods have been used for detection of protein-based toxins. The bacterium is usually the target of interest for clinical laboratories, the food industry, and, in the case of its toxins, for biodefense. In these specialized laboratories, immunoassay-based methods, molecular biology techniques, and mass spectrometry (MS) analysis through the application of matrix-assisted laser desorption/ionization (MALDI-TOF) identification, are applied [8]. The most widely used techniques for detecting clostridial toxins are enzyme immunoassay (EIA) and polymerase chain reaction (PCR). Although EIA is rapid and sensitive, cross reactivity could cause a high false positive rate that can lead to a misguided public health response. Additionally, clostridial toxins are unstable and readily degraded, which can lead to false negative results in cases when the samples are inappropriately stored or handled [9,10]. On the other hand, utilizing PCR also comes with difficulties, because many of the toxin genes in *Clostridium spp*. are placed on extrachromosomal elements (e.g., plasmids or phages) and can be horizontally transferred to other types of bacteria or even within *Clostridium* species. This transfer of genetic material can be problematic for methods targeting only one or a few genes from a single species [11,12,13]. Those disadvantages have been overcome by utilizing techniques of mass spectrometry coupled with liquid chromatography for multiplex detection of protein toxins, including *C. perfringens* toxins [14,15,16].

The main aim of the present study was to develop a targeted proteomic method for the detection of selected *C. perfringens* protein toxins. Due to a lack of commercially available protein toxin standards necessary for the methods development, recombinant toxins were first prepared. The developed detection and quantification assay is based on antibody-free sample preparation followed by bottom-up liquid chromatography with tandem mass spectrometry (LC-MS/MS) performed in targeted mode.

## 2. Results and Discussion

An unambiguous, multiplex, quantitative parallel reaction monitoring (PRM) assay for six bacterial protein toxins of the bacterium *C. perfringens* of potential warfare significance is reported. Mass spectrometry constitutes a sensitive and high-fidelity technology suitable for detecting selected toxin agents. This study contributes to improving that technology in this application. It should not be surprising that the application of MS, especially for food samples, has increased over the years. Targeted MS has been regarded as one of the most effective tools for quantitative analysis of proteins, and recently it has also been applied in clinical settings [17].

### 2.1. Growth Curve

**Figure 1:**
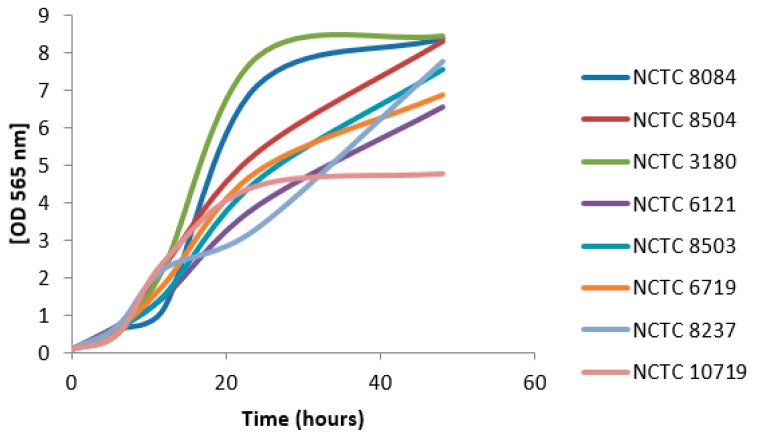
Growth curves for selected *Clostridium perfringens* strains.

Growth of the selected *C. perfringens* strains was examined at 37 °C in anaerobic conditions within commercially available BBL Fluid Thioglycolate Medium. This medium was selected with reference to the available publications [18,19]. Optical density was measured at five intervals for 48 h. Growth curves for selected strains of *C. perfringens* are shown in Figure 1. In accordance with the growth curves, the interval of 12 h was determined for subsequent analysis to assure that the cultures were not in the late stationary phase and not degraded by massive bacterial lysis.

### 2.2. Toxin Characterization and Identification of Unique Peptides

#### 2.2.1. Characterization of *C. perfringens* Proteins in Culture Filtrates and Whole-Cell Lysates

**Figure 2:**
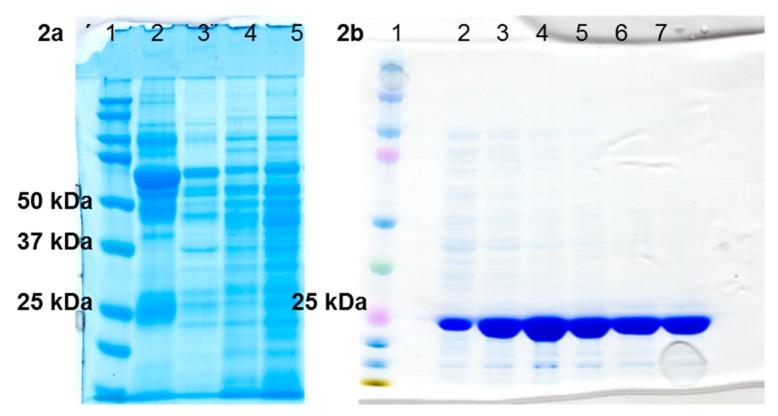
(**a**) Separation of *C. perfringens* proteins (strain NCTC 8237) from culture filtrates and whole-cell lysates via one-dimensional SDS-PAGE. Expected toxin production from this strain is alpha (MW = 45.5 kDa) and beta2 (MW = 31 kDa), 10 μg of protein sample per well. Well numbers 1–5: (1) kaleidoscope molecular weight standard; (2) Schaedler medium, culture filtrate; (3) Schaedler medium, whole-cell lysate; (4) thioglycolate medium, culture filtrate; (5) thioglycolate medium, whole-cell lysate. (**b**) SDS-PAGE of recombinant beta2 protein—fractions from His-tag purification, 10 μL of protein sample per well. Well numbers 1–7: (1) kaleidoscope molecular weight standard; (2) flow through; (3) wash; (4–7) E1–E4 elution fractions.

The strains were cultivated under anaerobic conditions in two different liquid media (Schaedler medium and Thioglycolate broth) to compare the presence of the observed toxins. The contents of protein toxins of interest in culture filtrates and in whole-cell lysates were examined simultaneously using data-dependent MS/MS analyses. An analysis summary of protein toxins produced by each selected *C. perfringens* strain is shown in Table 1. A detailed list of the detected peptides is summarized in Table S1. A representative one-dimensional sodium dodecyl sulfate (SDS) gel of these samples from one strain (NCTC 8237) is shown in Figure 2a. Due to the complexity of the protein samples prepared from natural producers and low production of the toxins of interest, it was unrealistic to use them for standard-grade toxins preparation for MS analysis without immunoaffinity enrichment using specific antibodies. Further experiments to obtain protein toxin standards have thus been directed to the preparation of protein toxins in a recombinant manner within *E. coli*.

Detection of the declared toxins was only partially successful in some of the studied strains. This probably was caused by the fact that production of some toxins may be limited to specific culture conditions [20]. On the other hand, in strains 13110 and 10719, beta2 toxin was detected to an unexpectedly large extent. This toxin was described in the 1990s [21]. It is probably produced by all *C. perfringens* strains [22], although it was not historically declared in the collection strains.

#### 2.2.2. Recombinant Protein Toxins from *C. perfringens*

The alpha, beta, beta2, epsilon, iota a, and iota b proteins of *C. perfringens* were expressed in *E. coli* NiCo21(DE3) and isolated through a polyhistidine tag in sufficient quantity and purity for MS analysis. One of the advantages of this approach is that the recombinant strains are available for preparation of new batches of standard proteins in case of need for further study or in case of suspected violent abuse of *C. perfringens* toxins. Identities and purities of the recombinant proteins were confirmed via one-dimensional electrophoresis (Figure 2b) and mass spectrometry. In-depth characterization of purified recombinant toxins was provided using data-dependent MS/MS analyses. Numbers of identified peptides and sequence coverages are listed in Table 2 (sequence coverages and a list of detected peptides are shown in Figure S1).

### 2.3. Multiplex Targeted LC-MS/MS Detection (PRM Method Development)

#### 2.3.1. Selection of Proteotypic Peptides

Selection of representative peptides for targeted protein quantification is critical for targeted proteomic workflow development. In the present proteomic assay, the target peptide sequences of the candidate proteins were selected based on such bioinformatics tools as Peptide Cutter, BLAST analysis, previous publications, and our own measured data. According to the criteria for target peptides selection and the data from shotgun analysis, two or three peptides were selected for each toxin. Their analogues, isotopically labeled synthetic peptides with labeled lysine (^13^C_6_^15^N_2_) or arginine (^13^C_6_^15^N_4_), were purchased for the optimization of PRM parameters, to check LC-MS/MS responses, and to allow for absolute quantification. Using Skyline software and experimental data from natural producers as well as from the recombinant standards, the characteristics required for final analysis (retention times, m/z, and charge states) were determined for each peptide. These were then exported in the form of an inclusion list (Table 3). Collision energy and charge state of selected peptides was uniform: 27 kV and 2^+^. The PRM method was designed to monitor all six toxins in one run, and the peptides were monitored for the whole duration of the MS analysis (not-scheduled mode).

#### 2.3.2. Calibration, Limits of Detection, and Quantification

**Figure 3:**
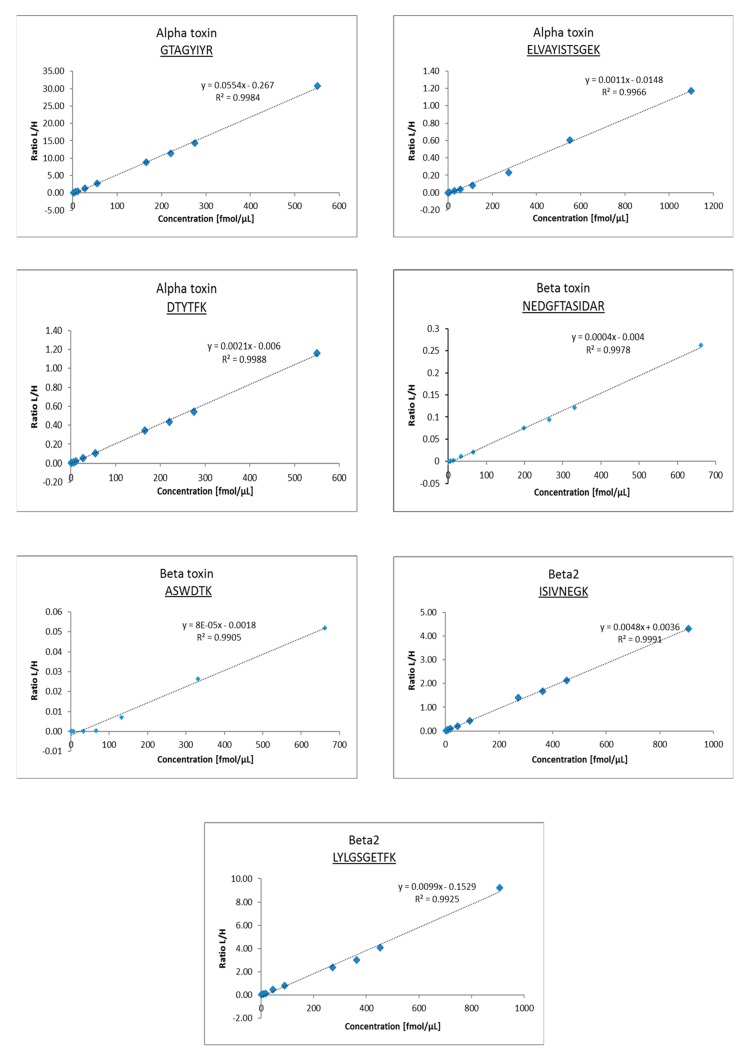
Calibration curves of selected peptides for each toxin.

**Figure 4:**
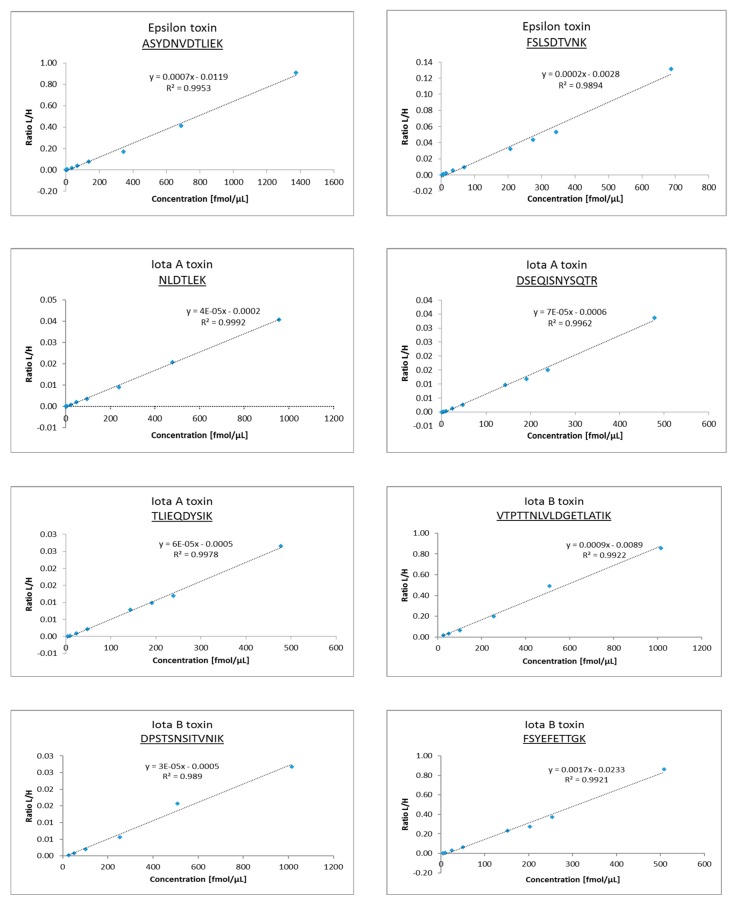
Calibration curves of selected peptides for each toxin.

Signals from labeled and unlabeled peptides eluting at the same time were acquired by the sequential acquisition method to avoid any overlap. The calibration curves were composed for each peptide in a range of concentrations from 0.635 fmol/μL to 1101 fmol/μL. Heavy peptides were added to standard digests always in the same concentration and used as internal standards. The measured data were further processed by Skyline software, and calibration lines were generated for each candidate peptide (Figure 3 and Figure 4). Lower limits of detection (LLOD) and quantification (LLOQ) were determined for each peptide (Table 4).

There exist multiple ways to determine LLOD and LLOQ [23,24]. In the present study, LLOD and LLOQ were determined by the method known as RSD (relative standard deviation) limit, which considers the variability of data at each point of the calibration curve.

The parameters of the acquired LLOQ and LLOD are shown in Table 4.

#### 2.3.3. Testing of the Developed PRM Method on Environmental Samples Supernatants from *C. perfringens* Cultures

Complex biological and environmental matrices contain various interferences, such as inorganic and organic compounds, high concentrations of competing antigens, and in cases of biological samples, also DNA. These result in considerable background signals and may cause false positive results. Primary testing of the developed detection method was here performed by two different approaches. First, various matrices spiked with the recombinant standards were extracted and analyzed by the PRM method. The matrices ranged from different kinds of inorganic material through simple organic matrix (milk) to the most complex ones as plant and animal tissues (see Supplementary Material and Results). These preliminary results showed strong absorption of protein toxins into the matrices (>90% in most peptides) which presents a substantial problem in the preparation of forensic samples. The most problematic matrix from this point of view was the animal tissue.

In the second part the method was applied also to culture supernatants of selected *C. perfringens* strains that were previously proven to have the presence of the toxin genes by PCR (data not shown). This approach was only partially successful because alpha and beta2 toxins were not detected by the targeted MS method at all (see Supplementary Material and Results). In alpha toxin it has been described previously that its production is highly dependent on specific culture conditions [20], and the stability of protein toxins in the culture media may be weak also due to degradation by proteases.

## 3. Conclusions

The present study describes a sensitive MS-based method for detecting and quantifying six protein toxins produced by *C. perfringens*. For the purpose of method development, all the toxins were prepared as recombinant standards in *E. coli* and isolated by a well-described affinity-tag protocol. This step is beneficial because the prepared recombinant strains can be used further and repeatedly for preparing standards in sufficient amounts and purity to enable relatively well-defined batch-to-batch reproducibility.

The targeted MS method described in this work was based on the PRM approach and utilizes the addition of synthetic heavy peptides that allow for quantification. For each protein, a set of two to three unique peptides was selected. Peptides from all six toxins can be thus analyzed together using a single inclusion list that can serve to monitor the potential presence of various *C. perfringens* toxinotypes in a single run. The presented detection limits ranged here in the order of fmol/µL to tens of fmol/µL, depending on the protein.

Testing of the developed PRM method on complex matrices revealed that the protein toxins tend to be strongly absorbed by the matrices. Especially the animal tissues present a substantial challenge due not only to the absorption but also to the high-protein background.

## 4. Materials and Methods

### 4.1. Safety Precautions

Due to the high toxicity, all experiments using *C. perfringens* cultures and their toxins were performed under biosafety level-2 conditions while following specified safety protocols and with special attention to personal protection equipment.

### 4.2. C. perfringens strains

Table 5 lists those strains used in the study and their expected production of toxins. Strains numbers 1–10 were obtained from the National Collection of Type Cultures (NCTC) of Public Health England, and number 11 is a clinical isolate from Pardubice Hospital in Pardubice, Czech Republic. Two strains were selected for each toxinotype.

### 4.3. Protein Standards Preparation

#### 4.3.1. *C. perfringens* Culture, Preparation of Culture Filtrates and Whole-Cell Lysates for Protein Characterization

Bacterial strains were revived and further cultivated in two liquid media: Schaedler Anaerobe Broth (CM0437, OXOID, Thermo Scientific, Waltham, MA, USA) and BBL Fluid Thioglycolate Medium (Becton Dickinson, Franklin Lakes, NJ, USA). Strains were cultivated overnight in anaerobic conditions at 37 °C within an anaerobic jar (Oxoid, AnaeroJar 2.5L, Thermo Scientific). The overnight culture was transferred to an agar plate (Schaedler Anaerobe Agar, CM0497 OXOID, Thermo Scientific) and cultivated overnight in anaerobic conditions at 37 °C within an anaerobic jar. A single isolated colony was transferred to both liquid media and cultivated overnight anaerobically. Culture supernatants and whole-cell lysates were prepared in accordance with Schwartz et al. [25]. Briefly, the bacterial suspensions were centrifuged for 5 min at 7000× *g* and 4 °C. The supernatants were filter-sterilized through a syringe-driven filter unit (0.22 µm, Merck, Darmstadt, Federal Republic of Germany), mixed with protease inhibitor cocktail (Complete EDTA-free, Roche) and stored at −20 °C for later processing. Cell pellets were suspended in 3 mL of 100 mM Tris-HCl buffer (pH 7.0), mixed with protease inhibitors then stored at −20 °C for 1 h. Subsequently, cells were sonicated on ice using an ultrasonic homogenizer (UP200St-G, Hielscher Ultrasonics, Teltow, Federal Republic of Germany) for 6 × 3 min at 20 kHz and 30 W and with 1.5 min cooling breaks. Unbroken cells were pelleted by centrifugation for 30 min at 10,000× *g* and 4 °C. Clear supernatants were transferred into new tubes. Unbroken cells were washed with 20 mM Tris-HCl buffer (pH 7.4) and centrifuged for 5 min at 10,000× *g* and 4 °C. The supernatants obtained from washing unbroken cells were mixed with the previous lysates. The final bacterial lysates were filter-sterilized as above. The whole-cell lysates as well as the culture filtrates were concentrated on 10 kDa Amicon Ultra-15 Centrifugal Filter Units (Merck) for 15 min at 7000× *g* and 4 °C, washed twice with 20 mM Tris-HCl buffer (pH 7.4), then concentrated to final volume. Protein concentration was determined by Pierce BCA Protein Assay Kit (Thermo Scientific). Ten micrograms of whole protein content from each sample was separated on one-dimensional SDS-PAGE (NuPAGE, Invitrogen, Waltham, MA, USA) and finally stained by Coomassie blue.

#### 4.3.2. Preparation of Recombinant Standard Toxins from *C. perfringens*

##### Bacterial Strains, Growth Conditions, Plasmids, and Primers

Bacterial strains, plasmids, and primers used in this work are listed in Table 6. *C. perfringens* NCTC 8237, NCTC 13110, and NCTC 8084 were used as donors of DNA. *C. perfringens* isolates were grown overnight under anaerobic conditions in Schaedler Anaerobe Broth and BBL Fluid Thioglycolate Medium at 37 °C.

*Escherichia coli* XL-1 Blue and NiCo21(DE3) (New England BioLabs, Ipswich, MA, USA) were used for DNA manipulation and toxins production, respectively. *E. coli* strains were grown at 37 °C aerobically on Luria-Bertani agar plates or in Luria-Bertani (LB) broth (Sigma Aldrich, St. Louis, MS, USA). Inducible production of proteins in *E. coli* NiCo21(DE3) was carried out in Terrific Broth (Merck) under shaking at 200 rpm. Kanamycin (50 µg/mL) was added to the medium. Enzymes for DNA cloning were supplied by New England BioLabs (NEB). Unless otherwise stated, all other reagents were from SERVA, Qiagen, or TaKaRa. All oligonucleotide primers were synthesized by Generi Biotech (Hradec Králové, Czech Republic). General genetic techniques for *Clostridium* and *E. coli* have been described previously [26,27].

##### Plasmid Construction

A set of plasmids containing the *C. perfringens plc*, *cpb*, *cpb2*, *etx*, *iap* and *ibp* genes was constructed as follows: Genomic DNA was isolated from *C. perfringens* cultures using the AquaPure Genomic DNA Isolation Kit (Bio-Rad) according to described protocols [26,28,29,30]. Fragments were amplified by PCR with flanking enzymes NcoI, XhoI, NdeI and XhoI (NEB) sites. The amplified fragments were inserted into NcoI, XhoI, NdeI and XhoI digested pET42b and pET28b vectors, respectively.

##### Expression of Recombinant Proteins

All proteins were expressed in the *E. coli* NiCo21(DE3) strain transformed with the expression plasmids pET28b+::plc, pET28b+::cpb2, pET42b+::cpb2, pET42b+::etx, pET42b+::iap and pET42b+::ibp under induction by 1 mM isopropyl-D-1-thiogalactopyranoside for 12 h at 37 °C. Cell pellets were disrupted by two passages in a French pressure cell at 16,000 psi in lysis buffer (50 mM NaH_2_PO_4_, 300 mM NaCl, pH 7.5), and subsequently clarified by centrifugation. Clarified lysates were purified on a charged Ni^2+^ chelating column (TALON^®^ Metal Affinity Resins, Clontech Laboratories, Mountain View, CA, USA) according to the described protocol [31]. Concentration of protein was determined using a bicinchoninic acid (BCA) assay kit (Pierce BCA Protein Assay Kit, Thermo Fisher Scientific). Recombinant proteins were separated by one-dimensional gel electrophoresis to prove the presence of protein of the appropriate molecular weight and purity. The identity of the recombinant proteins was confirmed by shotgun tandem mass spectrometry.

### 4.4. Growth Curves Measurement

Bacterial strains were revitalized and cultivated overnight in Schaedler Anaerobe Broth in anaerobic conditions at 37 °C. The overnight culture was transferred onto Schaedler Anaerobe Agar and cultivated overnight in anaerobic conditions at 37 °C. One isolated colony was transferred to BBL Fluid Thioglycolate Medium and all cultures were incubated at 37 °C in anaerobic conditions (Anaerobic & Microaerophilic Workstation, Baker Ruskinn) to reach optical density of 0.1 for each bacterial strain. Growth curves were generated for eight *C. perfringens* strains (NCTC 8084, 8504, 3180, 6121, 8503, 6719, 8237, and 10719). Measurement of optical density of bacterial suspensions was performed according to McFarland on a DEN-1 McFarland densitometer (Biosan, Riga, Latvia). Optical density was recorded at 0, 6, 12, 24, and 48 h and the curves were constructed and smoothed.

### 4.5. Preparation of Protein Samples for Mass Spectrometry Analysis

Samples were treated for MS analysis in accordance with in-gel and in-solution tryptic digestion, two main bottom-up proteomic approaches for preparation of protein samples [22]. For in-solution digestion, proteins were solubilized by 1 M urea, reduced by 10 mM tris(2-carboxyethyl)phosphine (37 °C, 45 min, 600 RPM), and alkylated by 10 mM iodoacetamide (room temperature, 10 min in darkness). The excess of iodoacetamide was quenched by 10 mM dithiothreitol at room temperature for 10 min. Sequencing-grade modified trypsin (Promega) was then added and the mixture was incubated overnight at 37 °C. The digestion process was stopped by the addition of trifluoroacetic acid (TFA), and the resulting peptides were desalted using phase extraction cartridges (Empore Solid Phase Extraction Cartridges, C18, Merck), according to the manufacturers’ protocol. Desalted peptides were vacuum-dried and stored at −20 °C until MS analysis. For in-gel digestion, the bands were manually excised and cut into small pieces. The gel pieces were destained using 100 mM Tris/HCl (pH 8.5) in 50% acetonitrile (ACN), then equilibrated in 50 mM ammonium bicarbonate (pH 7.8) in 5% ACN for 30 min at room temperature. They were then reduced by 100 mM tris(2-carboxyethyl)phosphine (room temperature, 30 min, 600 RPM) and alkylated by 300 mM iodoacetamide (room temperature, 30 min in darkness). Supernatants were removed, and the gels were vacuum-dried. Sequencing-grade modified trypsin in 50 mM ammonium bicarbonate (pH 7.8) in 5% ACN was then added to the dried gel pieces and the mixture was incubated at 37 °C and 600 RPM overnight. Peptides were then extracted using 70% ACN (60 °C, 30 min, 1000 RPM). The supernatants were transferred into new tubes. The gels were then extracted again using 100% ACN and vortexing. The supernatants were then combined. Samples were vacuum-dried and stored at −20 °C until MS analysis.

### 4.6. Internal Standards

SpikeTides™ Proteomics Peptide Standards (JPT Peptide Technologies) as stable isotope labeled peptides (heavy peptides) are customized peptides used as peptide standards for protein quantification for SRM/MRM and PRM assays. Isotope labeling was performed using [^13^C_6_^15^N_2_] L-lysine and [^13^C_6_^15^N_4_] L-arginine. JPT peptides selected for the targeted analysis are listed in Table 7.

### 4.7. Mass Spectrometry and Liquid Chromatography

LC-MS/MS analyses were performed on the UltiMate 3000 RSLC-nano System (Dionex, Sunnyvale, CA, USA) coupled on-line to a Q-Exactive mass spectrometer (Thermo Scientific). Peptide mixtures were dissolved in 2% ACN/0.05% TFA, loaded onto a capillary trap column (C18 PepMap100, 3 µm, 100 Å, 0.075 × 20 mm; Dionex) for preconcentration purposes, then separated on a capillary column (C18 PepMap RSLC, 2 µm, 100 Å, 0.075 × 150 mm; Dionex). Mobile phase A was 0.1% formic acid (*v*/*v*) and mobile phase B was 80% ACN/ 0.1% formic acid (*v*/*v*). The flow rate of the loading solvent was 200 μL/min for 5 min, and a constant flow rate of 300 nL/min was maintained during separation. The column was kept at 40 °C and the eluent was monitored at 215 nm.

### 4.8. Shotgun Analysis

Separation conditions included a step linear gradient of mobile phase B over mobile phase A from 4% to 36% B over 19 min and from 36% to 55% B through 6 min. The eluent was sprayed directly using an on-line coupled Nanospray Flex Ion source (Thermo Scientific) into a Q-Exactive mass spectrometer. The mass spectrometer was operated in the positive ion mode performing survey MS (at 350–1650 *m*/*z*) and data-dependent MS/MS scans of the seven most intense precursors, with a dynamic exclusion window of 23 s and isolation window of 1.6 Da. MS scans were acquired with resolution of 70,000 from 10^6^ accumulated charges. The maximum fill time was 100 ms. Normalized collision energy for HCD fragmentation was 27 units. MS/MS spectra were acquired with resolution of 17,500 from 10^5^ accumulated charges. The maximum fill time was 100 ms.

#### Protein identification

Representative MS/MS spectra from the *C. perfringens* toxins peptides selected for PRM analysis with fragment ion assignments are shown in Figure S2. A database search was performed using Proteome Discoverer software v. 1.4.1.14 (Thermo Scientific) and Mascot search algorithm. MS and MS/MS spectra were searched against a merged database consisting of the *C. perfringens* proteomes (*C. perfringens* strain 13; ATCC 13124; SM101; ATCC 3626; JGS1495; str. F4969; str. JGS1721; str. JGS1987; NCTC 8239; and WAL-14572). The merged database contained 32,700 sequences (downloaded on 18 May 2016). Search parameters were set as follows: tryptic specificity with maximum of 2 missed cleavages, cysteine carbamidomethylation as fixed modification and methionine oxidation as variable modification, mass tolerance of 10 ppm for precursors and 20 mmu for product ions. False discovery rate (FDR) was set to 0.01 (high confidence) and 0.05 (medium confidence).

The mass spectrometry proteomics data have been deposited to the ProteomeXchange Consortium via the PRIDE [32] partner repository with the dataset identifier PXD012528.

### 4.9. Targeted MS Analysis-Parallel Reaction Monitoring

Separation conditions included a step linear gradient of mobile phase B over mobile phase A from 4% to 34% B over 36 min and from 34% to 55% B through 11 min. A Q-Exactive mass spectrometer was operated in positive ion mode and was set for PRM acquisition. Survey MS scans (300–1500 *m*/*z*) were acquired with resolution of 35,000 from 3 × 10^6^ accumulated charges. Maximum fill time was 200 ms. MS/MS spectra were acquired from ions listed in the inclusion list in unscheduled mode, with resolution of 17,500 from 2 × 10^5^ accumulated charges. Maximum fill time was 60 ms, and the isolation window was 1.6 Da. Normalized collision energy for HCD fragmentation was 27 units.

#### 4.9.1. Selection of Peptides

Toxin sequences were obtained from the UniProt database (http://www.uniprot.org) for alpha, beta, beta2, epsilon, iota a, and iota b entries. Q0TV31, Q46308, Q5MQ79, Q02307, Q46220 and Q46221 were downloaded, respectively. Peptides for targeted assays were selected using Skyline software v. 4.1 (downloaded from https://skyline.ms/project/home/begin.view) [23]. The spectral library was created from data-dependent acquisition MS analyses of the recombinant proteins.

Skyline parameters for peptide settings were set as follows: enzyme trypsin with no missed cleavages; peptide length from 5 to 25 amino acids; methionine-containing peptides were excluded; cysteine carbamidomethylation was allowed; isotope label type was set up as heavy (^13^C_6_^15^N_2_ lysine and ^13^C_6_^15^N_4_ arginine); transitions were set from 2+ and 3+ precursor ions. For each targeted peptide, the Skyline result was manually inspected. For each toxin, two or three unique peptides were selected, showing the best signal intensity and chromatographic peak shape for a given parent protein. Peptides and their labeled counterparts were included in one common inclusion list.

#### 4.9.2. Calibration, Limits of Detection and Quantification

All the six recombinant protein toxins were mixed to form a standard protein mixture (10 μg of each protein and 20 μg of toxin I b) and then digested in solution. Heavy peptides were analyzed in amounts ranging from 10 to 5000 fmol. A final mixture for calibration was then prepared according to Table 8 in order to give the optimal MS signals for each individual heavy peptide (see Figure S3).

The calibration series was then prepared by sequential dilution of the standard protein mixture digest with mobile phase A and adding the same amount of heavy peptide mixture to each calibration sample. The resulting calibration samples were measured in triplicate in PRM mode with blank injections between each concentration to prevent possible carryover. Raw calibration data were processed in Skyline. Peak areas for all transitions were summed for each peptide to give the peptide peak areas. Ratios of corresponding light to heavy peptide peak areas were then processed in Excel to form the calibration curves and to calculate limits of detection and quantification.

LLOQ was defined as the lowest concentration with coefficient of variance (CV) of the triplicate <25%. LLOD was then calculated as LLOQ divided by 3. Linear regression was used to calculate the linearity of the response curve (over any three points of the curve) [24].

## Figures and Tables

**Table 1:** Detection of *C. perfringens* toxins in culture filtrates and whole-cell lysates of selected natural producers cultivated in two different media (a detailed list of detected peptides is in Table S1).

Strain Number (NCTC)	Expected Production of Protein Toxins	Number of Identified Peptides/Sequence Coverage (%)			
		Sch Medium Culture Filtrates	Tg Medium Culture Filtrates	Sch Medium Wcl	Tg Medium Wcl
8084	Alpha	3/16			
	Iota A				
	Iota B				
8504	Alpha				
	Epsilon		9/32	5/39	4/21
3180	Alpha	6/25			
	Beta	23/80	16/68		14/68
6121	Alpha				
	Beta		8/51	19/79	13/62
13110	Alpha				
	Beta	22/80	17/67	17/77	13/57
	Epsilon	6/33			
			Beta2 (11/54)		
8503	Alpha				
6719	Alpha				
	Iota A				
	Iota B				
Cl. P. A VK	Beta 2				
8237	Alpha	10/37	5/19	7/31	
	Beta 2				
10719	Alpha				
	Beta	16/56	11/56	22/73	12/61
		Beta2 (10/47)	Beta2 (9/39)	Beta2 (14/61)	Beta2 (11/41)
Pce	N/A	Alpha (10/40)			

Sch = Schaedler medium; TG = thioglycolate medium; WCL = whole-cell lysate; N/A = not available.

**Table 2:** Recombinant protein toxins and their mass spectrometry characterization (a detailed list of identified peptides is in Table S2).

Toxin	Number of Identified Peptides/Sequence Coverage (%)
Alpha	75/93
Beta	31/78
Beta2	36/78
Epsilon	72/92
Iota A	38/52
Iota B	94/63

**Table 3:** Inclusion list.

Peptide Sequence (Heavy Amino Acids Highlighted)	Precursor Ion *m*/*z* (as Targeted in Quadrupole)	Peptide Sequence (Heavy Amino Acids Highlighted)	Precursor Ion *m*/*z* (as Targeted in Quadrupole)
Protein name: Alpha		Protein name: Epsilon	
GTAGYIYR GTAGYIY**R** ELVAYISTSGEK ELVAYISTSGE**K** DTYTFK DTYTF**K**	450.732334 455.736469 648.837725 652.844824 387.687061 391.694161	ASYDNVDTLIEK ASYDNVDTLIE**K** SQSFTCK SQSFTC**K** FSLSDTVNK FSLSDTVN**K**	684.338089 688.345188 429.194728 433.201827 505.761289 509.768388
Protein name: Beta		Protein name: Iota A	
NEDGFTASIDAR NEDGFTASIDA**R** ASWDTK ASWDT**K**	648.296755 653.30089 354.171578 358.178678	NLDTLEK NLDTLE**K** DSEQISNYSQTR DSEQISNYSQT**R** TLIEQDYSIK TLIEQDYSI**K**	416.724175 420.731274 714.323501 719.327636 605.321711 609.32881
Protein name: Beta2		Protein name: Iota B	
ISIVNEGK ISIVNEG**K** LYLGSGETFK LYLGSGETF**K**	430.24782 434.24782 557.79259 561.79969	VTPTTNLVLDGETLATIK VTPTTNLVLDGETLATI**K** DPSTSNSITVNIK DPSTSNSITVNI**K** FSYEFETTGK FSYEFETTG**K**	943.527681 947.53478 688.356813 692.363912 604.777136 608.784235

**Table 4:** Summary evaluation of the response of quantitation peptides from selected toxins in blank matrix.

Toxin	Peptide	Lower Limit of Quantification (fmol/µL)	Lower Limit of Detection (fmol/µL)
Alpha	GTAGYIYR	1.38	0.46
	ELVAYISTSGEK	0.69	0.23
	DTYTFK	1.38	0.46
Beta	NEDGFTASIDAR	13.23	4.41
	ASWDTK	132.30	44.10
Beta2	ISIVNEGK	2.27	0.76
	LYLGSGETFK	2.27	0.76
Epsilon	ASYDNVDTLIEK	0.86	0.29
	FSLSDTVNK	6.87	2.29
Iota A	NLDTLEK	4.78	1.59
	DSEQISNYSQTR	1.20	0.40
	TLIEQDYSIK	23.90	7.97
Iota B	VTPTTNLVLDGETLATIK	25.40	8.47
	DPSTSNSITVNIK	25.40	8.47
	FSYEFETTGK	5.00	1.69

**Table 5:** Strains of *C. perfringens*, expected production of protein toxins, presence of toxin genes and their localization.

Number	Strain Number (NCTC)	Expected Production of Protein Toxins	Accession Number of Protein Toxins	Presence of Toxin Genes	Localization of Gene
1 *	8084	Alpha	Q0TV31	plc	Chm
		Iota A	Q46220	iap	Plasmid
		Iota B	Q46221	ibp	Plasmid
2	8504	Alpha	Q0TV31	plc	Chm
		Epsilon	Q02307	etx	Plasmid
3	3180	Alpha	Q0TV31	plc	Chm
		Beta	Q46308	cpb	Plasmid
4	6121	Alpha	Q0TV31	plc	Chm
		Beta	Q46308	cpb	Plasmid
5 *	13110	Alpha	Q0TV31	plc	Chm
		Beta	Q46308	cpb	Plasmid
		Epsilon	Q02307	etx	Plasmid
6	8503	Alpha	Q0TV31	plc	Chm
7	6719	Alpha	Q0TV31	plc	Chm
		Iota A	Q46220	iap	Plasmid
		Iota B	Q46221	ibp	Plasmid
8	Cl. P. A VK	Beta 2	Q5MQ79	cpb2	Plasmid
9 *	8237	Alpha	Q0TV31	plc	Chm
		Beta 2	Q5MQ79	cpb2	Plasmid
10	10719	Alpha	Q0TV31	plc	Chm
		Beta	Q46308	cpb	Plasmid
11	Pce	N/A			

NCTC = National Collection of Type Cultures of Public Health England; Pce = Clinical isolate from Pardubice Hospital; N/A = not available; Chm = chromosomal gene location. * Strains that were further used for DNA isolation for recombinant standards preparation.

**Table 6:** Bacterial strains, plasmids, and primers used in this study.

Strains, Plasmids, or Primers	Description	Source or Reference
***C. perfringens* strains**		
NCTC 8237	Toxinotype A Isolated from: human, bovine	Culture Collections, Public Health England
NCTC 13110	Toxin status: Epsilon toxin producer Isolated: N/A	Culture Collections, Public Health England
NCTC 8084	Toxinotype E Isolated from: mammal, intestinal tract of a calf	Culture Collections, Public Health England
***E. coli* strains**		
*XL-1 Blue*	F’*::Tn10 proA^+^B^+^ lacI^q^ Δ(lacZ)M15/ recA1 endA1 gyrA96* (Nal^R^) *thi hsdR17 (rK^–^ mK^+^) glnV44 relA1 lac*	Stratagene
NiCo21(DE3)	*can::CBD fhuA2 [lon] ompT gal (λ DE3) [dcm] arnA::CBD slyD::CBD glmS6Ala ∆hsdS λ DE3 = λ sBamHIo ∆EcoRI-B int::(lacI::PlacUV5::T7 gene1) i21 ∆nin5nechat*	New England BioLabs
**Plasmids**		
pET28b	*E. coli* protein expression vector, Km^R^	Novagen, Merck
pET28-plc	pET28b+::*plc*, Km^R^	this study
pET28-cpb	pET28b+::*cpb*, Km^R^	this study
pET42-cpb2	pET42b+::*cpb2*, Km^R^	this study
pET42-etx	pET42b+::*etx*, Km^R^	this study
pET42-iap	pET42b+::*iap*, Km^R^	this study
pET42-ibp	pET42b+::*ibp*, Km^R^	this study
**Oligonucleotides**	**Sequence (5′ to 3′)**	
alpha_FW_NcoI alpha_REV_XhoI	CCACCATGGATAAAAGAAAGATTTGTAAGGCGCTA TGGCTCGAGTTTTATATTATAAGTTGAATTTCCTGA	Generi Biotech
beta_FW_NdeI beta_REV_XhoI	CCACATATGAAGAAAAAATTTATTTCATTAGTTA TGGCTCGAGAATAGCTGTTACTTTGTGAGTAAG	Generi Biotech
Cpb2 Fw -NcoI 58C Cpb2 Rev -XhoI 58C	CGCGCCATGGATAATGAAGTGAATAAATACCAATC CGCGCTCGAGATAACAATAACCCTCACCAAAT	Generi Biotech
epsilon_FW_NdeI epsilon_REV_XhoI	CCACATATGAAAAAAAATCTTGTAAAAAGTTTAG TGGCTCGAGTTTTATTCCTGGTGCCTTAATATAAA	Generi Biotech
iota1a_FW_NdeI iota1a_REV_XhoI	AATCAAAATGAAATTTCTTTAGAGAAAT TGGCTCGAGATTTATCAATGTTGCATCCAAAATTA	Generi Biotech
iota1b_FW_NdeI iota1b_REV_XhoI	CCACATATGAATATACAAATTAAAAATGTATTTAG TGGCTCGAGATTAACACTAAGCACTAATAACTCT	Generi Biotech

**Table 7:** Selected isotopically labeled peptides from protein toxin standards.

Isotopically Labeled Peptides	Protein Toxin Standard
GTAGYIYR ELVAYISTSGEK DTYTFK	Alpha
NEDGFTASIDAR ASWDTK FTETTR	Beta
ISIVNEGK LYLGSGETFK	Beta 2
ASYDNVDTLIEK SQSFTC FSLSDTVNK	Epsilon
NLDTLEK DSEQISNYSQTR TLIEQDYSIK	Iota A
VTPTTNLVLDGETLATIK DPSTSNSITVNIK FSYEFETTGK	Iota B

**Table 8:** Composition of the mixture of all heavy peptides.

Protein	Peptide	Concentration in the Spike (fmol/μL)
alpha	GTAGYIYR	400
	ELVAYISTSGEK	400
	DTYTFK	400
beta	NEDGFTASIDAR	400
	ASWDTK	1000
beta2	ISIVNEGK	400
	LYLGSGETFK	400
epsilon	ASYDNVDTLIEK	400
	SQSFTCK	1000
	FSLSDTVNK	400
iota A	NLDTLEK	400
	DSEQISNYSQTR	400
	TLIEQDYSIK	400
iota B	VTPTTNLVLDGETLATIK	1000
	DPSTSNSITVNIK	1000
	FSYEFETTGK	400

## Supplementary Materials

The following are available online at https://www.mdpi.com/2072-6651/11/3/177/s1, Table S1: Detailed list of detected peptides of *C. perfringens* toxins in culture filtrates and whole cell lysates of selected natural producers cultivated in two different media. Table S2: Detailed list of identified peptides from recombinant protein toxins. Figure S1: Sequence coverages from LC-ESI-HRMS and MS/MS (DDA experiments) for the six recombinant toxins (100 ng of each/load). Sequence coverages of 93% for alpha toxin, 78% for beta toxin, 78% for beta2 toxin, 92% for epsilon toxin, 52% for Ia toxin and 63% for Ib toxin were obtained considering peptides detected with maximal 2 missed cleavages (highlighted). Figure S2: Representative MS/MS spectra from the *C. perfringens* toxins peptides selected for PRM analysis with fragment ion assignments (MS/MS spectra were obtained from Proteome Discoverer v. 1.4.1.14). Figure S3: Areas under curves of selected heavy peptides. Supplementary Methods and Results: Testing of the PRM method on individual environmental samples and culture filtrates of *C. perfringens* strains.
