# Supplementary material for: Targeted Mass Spectrometry Analysis of Clostridium perfringens Toxins
Source: Toxins (Basel). 2019 Mar 23;11(3):177. doi: 10.3390/toxins11030177 (PMC6468457; doi:10.3390/toxins11030177)
Supplement: Supplementary file 1 [file toxins-11-00177-s001.zip › toxins-465998-SI/Supplementary materials/toxins-465998-Supplementary Methods and Results.docx]

Supplementary Materials: Targeted Mass Spectrometry Analysis of *Clostridium perfringens* Toxins

Miloslava Duracova, Jana Klimentova, Alena Myslivcova Fucikova, Lenka Zidkova, Valeria Sheshko, Helena Rehulkova, Jiri Dresler and Zuzana Krocova

1. Testing of the PRM Method on Individual Environmental Samples

Six different matrices were used for targeted MS method evaluation. Sandy loam-soil (obtained in Hradec Králové—Březhrad, Czech Republic), peat (20 L pack, AGRO CS a.s., Czech Republic), and substrate for cacti (purchased in a local supermarket) were dried to a constant weight at 40 °C, homogenized in a mortar and sieved through a 2.5 mm mesh. Dried milk (Bohemilk 400 g, whole milk, manufactured by Bohemilk, a.s., Czech Republic) was used without further treatment. Homogenized animal tissue—one BALB/c mouse was killed in CO_2_ atmosphere, frozen to −80 °C and then homogenized in a particle mixer to a maximum particle size of 2.5 mm, after thawing it was used without further treatment. Experiments on mice were conducted under supervision of the institution’s Animal Unit and were approved by the Animal Care and Use Committee of the Faculty of Military Health Sciences, University of Defense, Hradec Kralove, Czech Republic. Spinach (cut spinach, frozen, AGRIMEX Vestec a.s., Czech Republic) was used without further processing after defrosting.

The matrices (50 mg of each) were artificially contaminated as follows: Standard protein mixture (see 4.9.2.) was spiked directly into an aliquot of each matrix to soak up the liquid, then incubated at 4 °C for 45 min without shaking. The samples were then suspended in 500 μL of deionized water and gently mixed at 4 °C for 50 min. Subsequently, samples were filtered through vacuum-driven sterile 20 μm filters, (Merck, Darmstadt, Federal Republic of Germany) to get rid of large particles. The filtrates were further centrifuged for 20 min at 62,000× g and 4 °C. Protein contents of the supernatants were determined by BCA. Exact amounts of the samples were digested in solution. Dried samples were dissolved in mobile phase A, mixed with heavy peptide mixture, and measured as described in 4.9. A standard protein mixture untreated by any matrix was measured in the same batch. The concentrations detected in matrix-treated samples were related to the concentrations in untreated standards and expressed as percentages thereof (Table 1).

**Table 1.** Concentrations detected in matrix-treated samples related to the concentration in untreated standard expressed in percent.

| **Matrices** | **Homogenized Animal Tissue** | **Dry Milk** | **Spinach** | **Sandy-Loam Soil** | **Substrate for Cacti** | **Peat** |
| --- | --- | --- | --- | --- | --- | --- |
| **Toxin**  **Peptide** |  |  |  |  |  |  |
| Alpha  GTAGYIYR  ELVAYISTSGEK  DTYTFK | 0  0  0 | 0  0  0 | 1.22  2.21  1.89 | 0  <1  1.4 | 5.64  <1  3.88 | <1  <1  <1 |
| Beta  NEDGFTASIDAR | 0 | 18.14 | 5.30 | 0 | 8.85 | 0 |
| Beta2  ISIVNEGK  LYLGSGETFK | <1  0 | 17.82  35.33 | 54.33  23.33 | 1.73  2.23 | 21.71  27.30 | <1  1.52 |
| Epsilon  ASYDNVDTLIEK  FSLSDTVNK | 7.33  3.87 | 6.59  5.84 | 5.81  5.30 | <1  <1 | 4.65  3.33 | <1  1.02 |
| Iota A  NLDTLEK  DSEQISNYSQTR  TLIEQDYSIK | 0  0  5.53 | 0  0  8.68 | 37.39  9.25  13.53 | 2.29  1.70  <1 | 30.18  12.08  8.81 | 0  0  0 |
| Iota B  VTPTTNLVLDGETLATIK | 0 | 0 | 39.14 | 3.73 | 0 | 0 |

2. Testing of the PRM Method on Culture Filtrates of *C. perfringens* Strains

Strains were cultivated overnight in anaerobic conditions at 37 °C in an anaerobic jar (Oxoid, AnaeroJar 2.5L, Thermo Scientific, Waltham, MA, USA). The overnight culture was transferred onto Schaedler Anaerobe Agar (CM0497 OXOID, Thermo Scientific) and cultivated overnight in anaerobic conditions at 37 °C within an anaerobic jar. One isolated colony was transferred to liquid media (BBL Fluid Thioglycolate Medium, Becton Dickinson, Franklin Lakes, NJ, USA) and cultivated overnight anaerobically. Subsequently, 9 mL of cultures were collected and centrifuged for 5 min at 7000× g and 4 °C. Culture supernatants were mixed with protease inhibitor cocktail (Complete EDTA-free, Roche), centrifuged on 10 kDa Amicon Ultra-15 Centrifugal Filter Units (Merck) for 15 min at 7000× g and 4 °C, washed twice with 20 mM Tris-HCl (pH 7.4), then concentrated to final volume. Protein concentration was determined by Pierce BCA Protein Assay Kit (Thermo Scientific, Waltham, MA, USA). Three hundred micrograms of proteins from each sample were digested in solution. The dried samples were dissolved in mobile phase A, mixed with heavy peptide mixture, and measured as described in 4.9. Concentrations of each peptide respectively produced by various *C. perfringens* strains were determined through calibration curves (Table 2).

**Table 2.** Concentrations of toxins detected by PRM method in culture filtrates of selected *C. perfringens* strains.

| **Toxin**  **Peptide** | **Strains (Measured Concentration in Culture Filtrate, fmol/µL)** | | |
| --- | --- | --- | --- |
| **Alpha** | **n. d.** |  |  |
| Beta |  |  |  |
| NEDGFTASIDAR | NCTC 3180 (1859.93) | NCTC 6121 (166.12) | NCTC 10719 (828.84) |
| Epsilon |  |  |  |
| ASYDNVDTLIEK  FSLSDTVNK | NCTC 8504 (37.46)  NCTC 8504 (704.76) |  |  |
| Iota A |  |  |  |
| DSEQISNYSQTR | NCTC 8084 (28.68) |  |  |
| TLIEQDYSIK | NCTC 8084 (85.37) |  |  |
| Iota B |  |  |  |
| FSYEFETTGK | NCTC 8084 (52.19) |  |  |
| Beta2 | n. d. |  |  |

n. d. not detected in any culture filtrate.
